# Neoadjuvant treatment of Dermatofibrosarcoma Protuberans of pancreas with Imatinib: case report and systematic review of literature

**DOI:** 10.1186/2045-3329-4-8

**Published:** 2014-08-06

**Authors:** Mashaal Dhir, David G Crockett, Todd M Stevens, Peter T Silberstein, William J Hunter, Jason M Foster

**Affiliations:** 1 Department of Surgery, Surgical Oncology, 986345 University of Nebraska Medical Center, Omaha, NE 68198-6345, USA; 2 Department of Hematology and Oncology, University of Nebraska Medical Center, Omaha, NE, USA; 3 Department of Pathology, University of Missouri, Kansas City, MO, USA; 4 Department of Hematology and Oncology, Creighton University Medical Center, Omaha, NE, USA; 5 Department of Anatomic Pathology, Creighton University Medical Center, Omaha, NE, USA

**Keywords:** Pancreatic metastases, DFSP, Imatinib, Systematic review

## Abstract

Dermatofibrosarcoma Protuberans (DFSP) is a rare skin tumor, characterized by frequent local recurrence but is seldom metastatic. It is histologically characterized by storiform arrangement of spindle cells. Cytogenetically, most tumors are characterized by translocation 17:22 leading to overexpression of tyrosine kinase PDGFB which can be targeted with tyrosine kinase inhibitor, Imatinib. We describe the first case of unresectable pancreatic metastases from DFSP treated with neoadjuvant Imatinib and subsequently R0 metastectomy. Additionally, a comprehensive systematic review of DFSP pancreatic metastases and the current published data on the use of Imatinib in DFSP is summarized.

## Background

Dermatofibrosarcoma Protuberans (DFSP) is a rare fibrohistiocytic tumor of the skin or subcutaneous tissue that is often locally infiltrative but rarely metastatic [1,2]. Cytogenetically, DFSP is characterized by a pathognomonic translocation t(17;22) (22;q13) with fusion of the COL1A1 gene on chromosome 17 with the PDGFB gene on chromosome 22 [3]. This event results in constitutive expression of ligand PDGFB creating an autocrine stimulatory loop that drives cell proliferation and fibrosis. Clinically, it commonly presents in younger adults growing slowly over many years [2,4]. Microsatellite instability and p53 mutations are involved in tumor progression to the fibrosarcomatous variant (DFSP-FS) [5]. Although the major recurrence risk for DFSP is local relapse, DFSP-FS subtype is associated with an aggressive clinical course, more likely to develop distant metastases [6,7]. Several case reports in the literature have demonstrated that DFSP can metastasize to the lungs [8-10], as well as, pancreatic or retroperitoneal spaces similar to the current case [11-13]. The optimal treatment of primary and metastatic DFSP is complete surgical resection with negative margins [14]. Over 90% of DFSPs, demonstrate a constitutive activation of platelet-derived growth factor receptor, making inhibition with a promiscuous tyrosine kinase inhibitor (TKI), Imatinib a good option for unresectable, recurrent, or metastatic disease [15]. There have been several reports of neoadjuvant Imatinib for locally advanced primary tumors and adjuvant Imatinib for resected margin positive primary disease and metastatic disease [16-20]. There have been no published reports of neoadjuvant Imatinib for unresectable metastatic DFSP to the pancreas successfully treated and subsequently resected. Here, we present the first reported case of unresectable metastatic DFSP to the pancreas, successfully resected following neoadjuvant Imatinib. Additionally, we conducted a systematic review of the literature for pancreatic metastases of DFSP and use of Imatinib in DFSP.

## Case presentation

**Figure 1:**
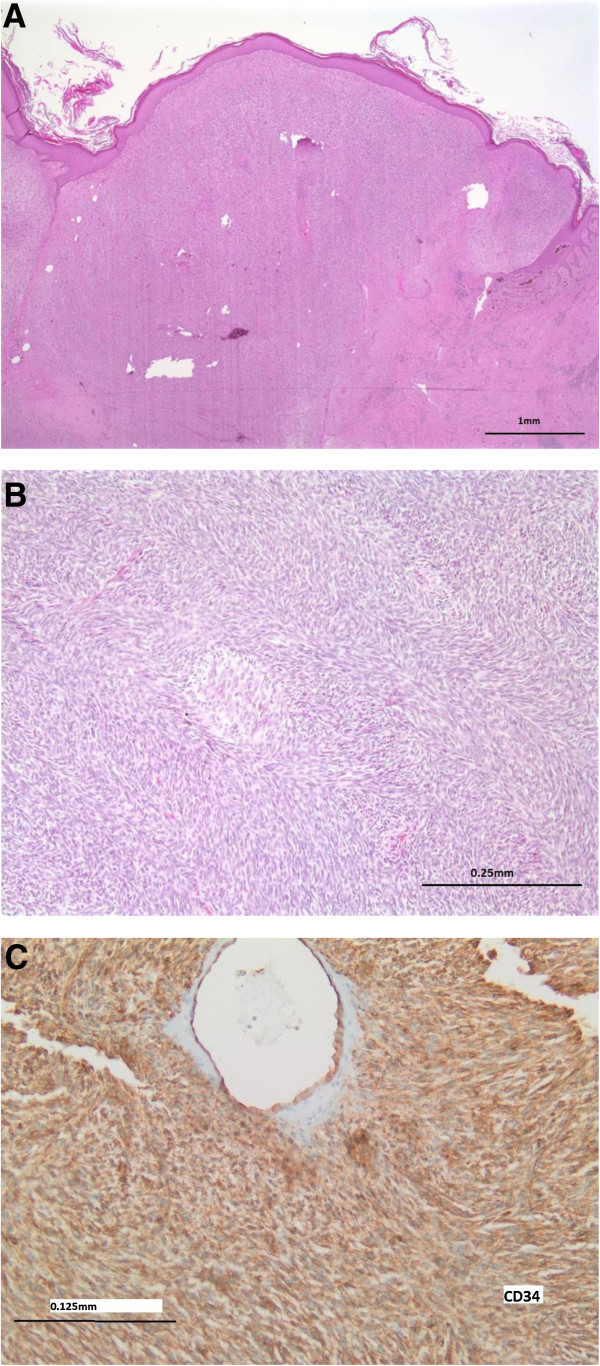
**Histology of Primary tumor. A)** The left chest wall mass excised in 2005 showed an invasive spindle cell neoplasm with **B)** areas of high grade, fibrosarcomatous transformation with Herringbone architecture. **C)** The tumor showed CD34 expression and this, along with the fibrosarcomatous areas and high mitotic rate, were consistent with a dermatofibrosarcoma protuberans with fibrosarcomatous transformation (DFSP-FS).

**Figure 2:**
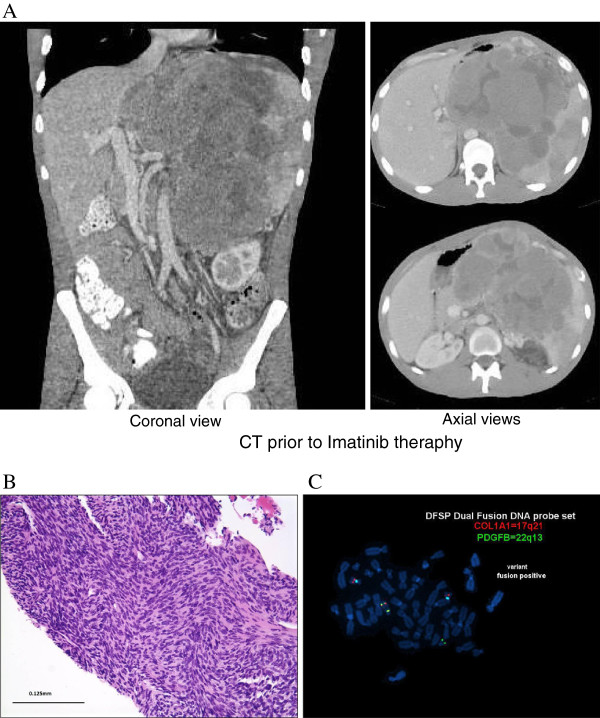
**Radiographs of metastatic disease at diagnosis. A)** CT scan demonstrating a large unresectable tumor involving all organs in the left upper abdomen. **B)** Biopsy of this mass showed malignant high grade spindled cells, **C)** a COL1A1-PDGFB translocation by dual fusion FISH probe set (yellow signals indicate the fusion signal), consistent with metastatic dermatofibrosarcoma protuberans with fibrosarcomatous transformation (DFSP-FS).

**Figure 3:**
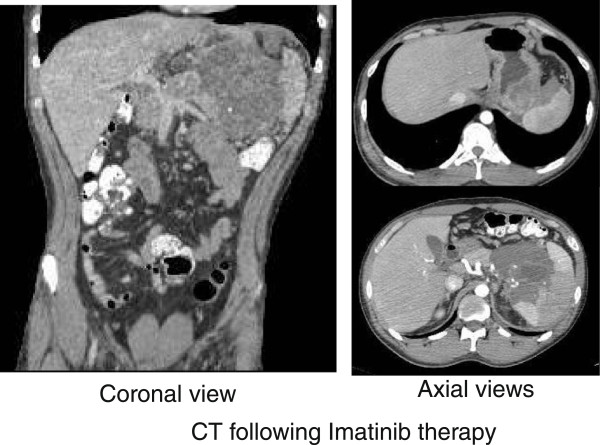
CT scan illustrating post imatinib decrease in size of the tumor now amenable to surgical resection.

**Figure 4:**
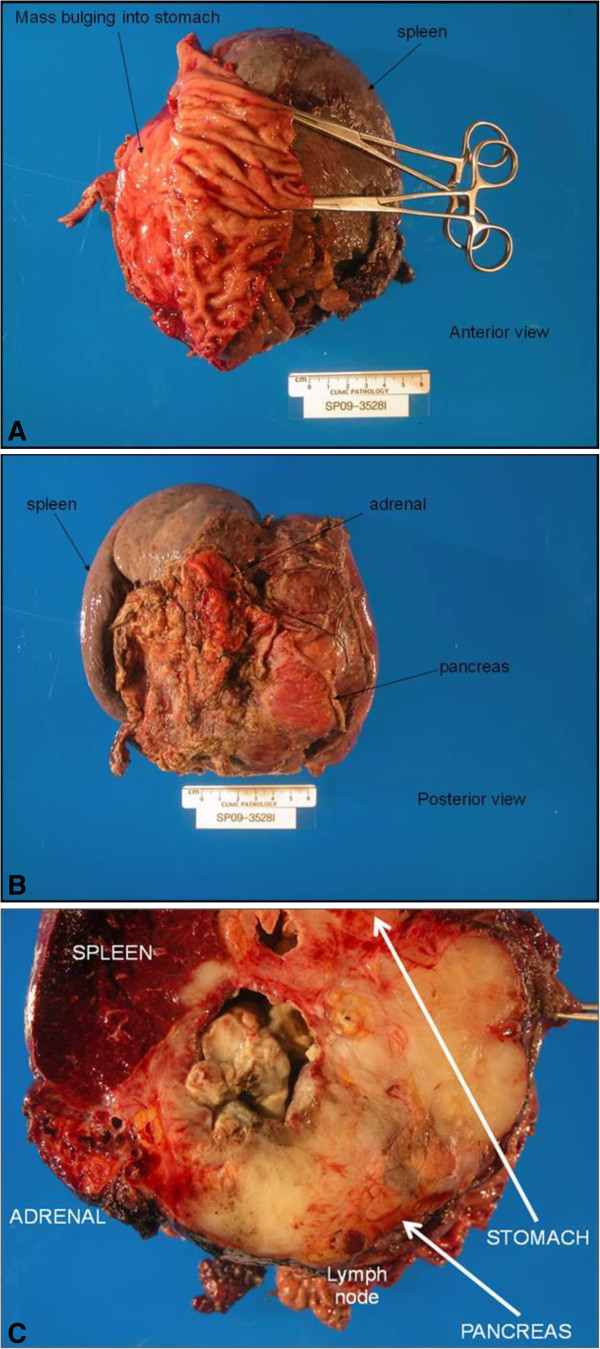
**Gross R**_**0 **_**tumor resection specimen: subtotal distal pancreatectomy, subtotal gastrectomy, splenectomy, adrenalectomy. A)** Anterior view **B)** Posterior view **C)** Cross sectional view.

**Figure 5:**
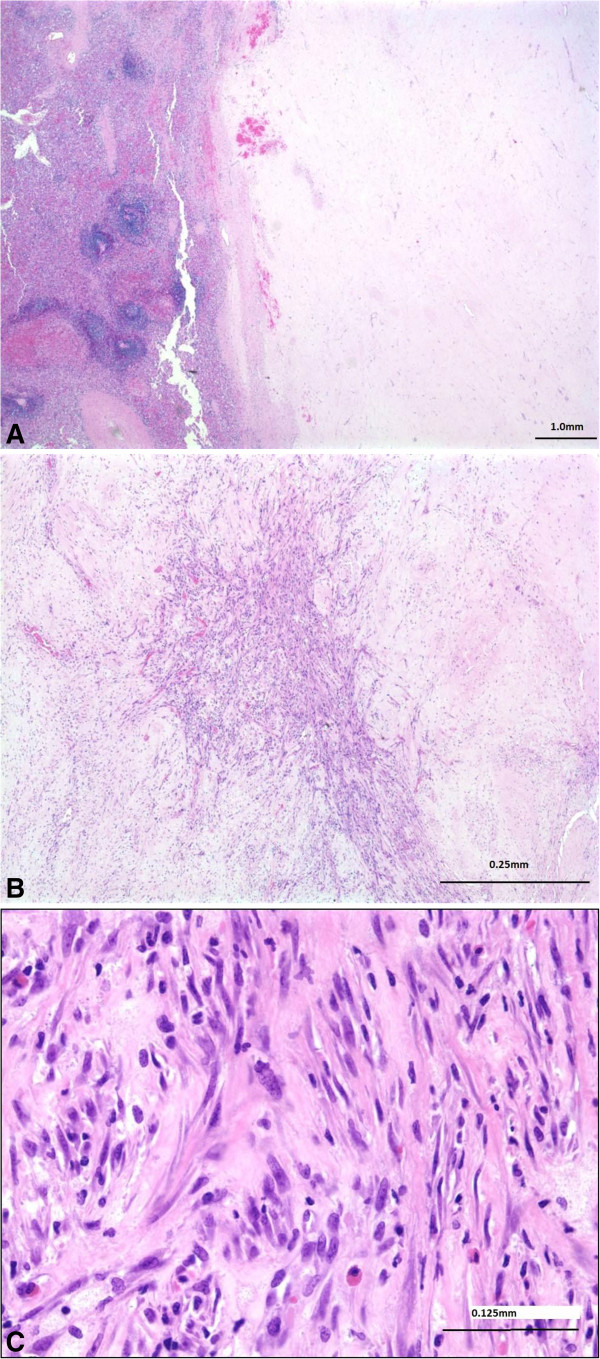
**Histology post-Imatinib Resected retroperitoneal tumor showed a hyalinized hypocellular mass. A)** Splenic tissue is demonstrated on the left in the current section. **B & C)** Focally, the tumor demonstrated residual atypical spindle cells that revealed a COL1A1-PDGFB fusion by interphase FISH (not shown), consistent with a dramatic histologic response of metastatic DFSP-FS to Imatinib.

A 31 year old African American male was diagnosed with DFSP of the skin and soft tissue of the anterior left chest wall and shoulder in 2005 and underwent local excision at an outside facility in December, 2005. Grossly, the excised specimen consisted of a 22.0 × 18.0 × 9.5 cm portion of skin & subcutaneous tissue that contained a multinodular and glistening dermal & subcutaneous mass. Histologically, this revealed a neoplasm consisting of atypical spindled cells arranged in a storiform pattern that invaded the dermis and subcutaneous tissue (Figure 1A). Large parts of this tumor showed areas histologically reminiscent of fibrosarcoma (at least 75% of the tumor), characterized by spindled mesenchymal cells with high grade cytologic atypia arranged in broad fascicles forming a “Herringbone” pattern (Figure 1B), with more than 10 mitotic figures per high powered field. This neoplasm showed expression of CD34 by immunohistochemistry (Figure 1C), but was negative for Desmin, S100, Pan-cytokeratin, BCL-2, and CD117 expression. The deep margin of resection was focally positive for the neoplasm. A diagnosis of dermatofibrosarcoma protuberans with fibrosarcomatous transformation (DFSP-FS) was rendered.The patient received no other therapy at that time and did well until October 2007, when he presented to the emergency department with severe anemia and a hemoglobin of 4 g/dL. Surgical Oncology was consulted after an abdominal CT scan revealed a heterogeneous mass in the left upper quadrant involving the pancreas, spleen, adrenal, left lobe of liver, and stomach measuring 17 × 18 × 23 cm (Figure 2A). A core biopsy of this retroperitoneal mass was obtained that showed spindle shaped mesenchymal cells with high grade cytologic atypia (Figure 2B) essentially identical to the fibrosarcomatous areas that were seen in the 2005 chest wall DFSP-FS. These malignant cells showed an identical immunophenotype as the original chest wall tumor (see above). A dual fusion DNA FISH probe set for the COL1A1 (17q21) and PDGFB (22q13) genes was positive for a COL1A1-PDGFB translocation (Figure 2C), consistent with DFSP-FS metastasis to the retroperitoneum.Radiographically, this retroperitoneal tumor was unresectable and the patient was started on Imatinib therapy 400 mg BID. The patient tolerated this dose without any reported side effects and after 18 months of Imatinib therapy, the tumor exhibited a dramatic response with a 70% size reduction and radiographically appeared to be amenable to margin negative (R0) surgical resection (Figure 3). A left upper quadrant exenteration (en-bloc resection including a subtotal distal pancreatectomy, splenectomy, left adrenalectomy, and subtotal gastrectomy) was performed with negative margins on September 1, 2009 (Figure 4A-C). Post-Imatinib, this resected tumor showed dramatically altered histology, revealing primarily hyalinized and myxoid fibrous differentiated stroma (Figure 5A) with focal atypical spindle cells (Figure 5B-C) that revealed a COL1A1-PDGFB fusion by interphase FISH analysis, consistent with a dramatic gross and histologic response of the metastatic DFSP-FS to the Imatinib. The lymph nodes were negative for tumor.

The patient resumed adjuvant Imatinib for 6 months and remained free of disease for 14 months, off therapy. His imaging at 14 months revealed para-aortic nodal disease without evidence of local recurrence that responded to reinstitution of Imatinib at the same dose. The patient is now five years out from the initial diagnosis of metastatic disease and remains in remission on Imatinib.

### Systematic review: Pancreatic metastasis of Dermatofibrosarcoma Protuberans

#### Methodology

A Medline search was performed using the key words (a) “Pancreatic metastasis” AND “Dermatofibrosarcoma Protuberans” (b) “Pancreas” AND “Dermatofibrosarcoma Protuberans”. Similarly, an Embase search was performed using the key words (a) “Pancreatic metastasis” AND “Dermatofibrosarcoma Protuberans”. Medline and Embase search using strategy (a) revealed 2 articles [13,21]. Search strategy (b) identified 3 articles [11,13,22]. Backward search was performed using the references from full texts of these 4 articles and no additional articles were found.

#### Results

We identified 4 articles reporting pancreatic metastases from DFSP [11,13,21,22]. A summary of these articles is provided in Additional file 1. Two of these patients were found to have pancreatic metastases during follow up and this appeared to be the only site of metastases [13,21]. In another patient reported by Winter et al., diagnosis was not clear preoperatively [11]. Interestingly, all three of these patients had aggressive disease with fibrosarcomatous variant of DFSP, high mitotic counts, relatively short disease free interval and did not have local recurrence prior to development of metastatic disease. In the report by Onoda et al. a patient had multiple local recurrences with relatively more aggressive features at each recurrence and finally died of brain metastases. Patient was found to have pancreatic and other systemic metastases at autopsy. No studies described the use of Imatinib as a neoadjuvant strategy to treat the pancreatic metastases.

### Systematic review: use of Imatinib in Dermatofibrosarcoma Protuberans

#### Methodology

**Figure 6:**
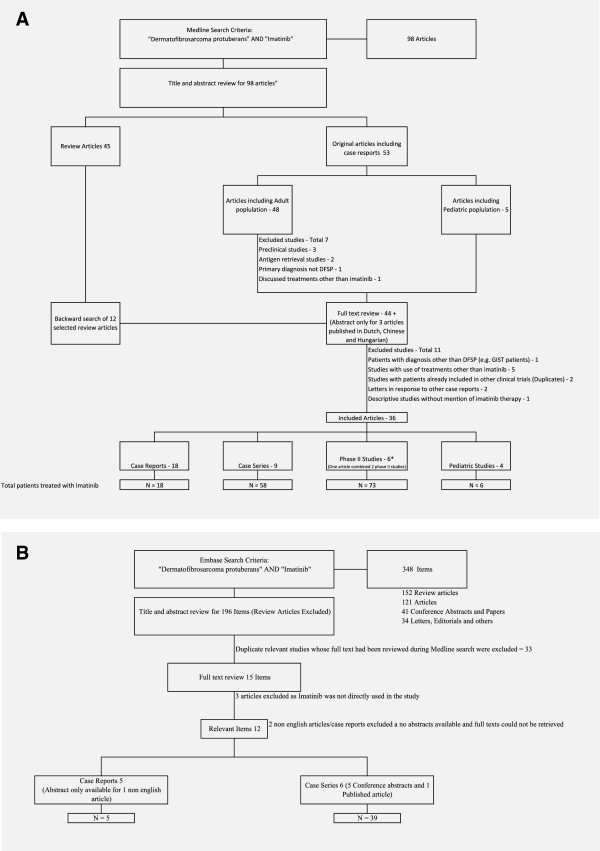
**Search strategy flow diagrams. A** &**B** Diagrams depicting the search strategy for systematic review of the use of Imatinib in patients with Dermatofibrosarcoma Protuberans.

Figure 6 describes the search strategy in detail. A Medline search was performed using key words “Imatinib” AND “Dermatofibrosarcoma Protuberans” which resulted in 98 articles. A title and abstract review (wherever available) of all articles was performed. After exclusion of preclinical studies (studies on cell lines) we were able to identify 48 articles which mentioned DFSP and either implied or reported the use of Imatinib in patients. Full texts of these 45 articles were reviewed. Information was gathered from abstracts only, for 3 studies in non-English languages. Backward search was also performed using the references of these 45 articles and full texts of 12 randomly selected review articles. Twelve studies were excluded (exclusion criteria mentioned in Figure 6). Briefly, exclusion criteria include a) studies which did not use Imatinib b) duplicate studies c) studies which discussed molecular characteristics only d) critiques of other case reports e) described patients whose primary diagnosis was not DFSP. A total of 36 articles were included in the systematic review [16,18-20,23-54]. Response to Imatinib was defined as complete clinical or pathologic response, partial response or stable disease.

After completing the Medline search, an Embase search was also performed using key words “Imatinib” AND “Dermatofibrosarcoma Protuberans” which revealed 348 items including 152 review articles. After exclusion of studies already identified using Medline search, we were able to identify an additional 5 case reports [55-59] and 6 case series [60-65] (5 in abstract form [60,61,63-65]). There was one more case report in non-English language, describing neoadjuvant Imatinib for DFSP of the face, for which no abstract was available [66].

#### Results

We identified 23 case reports [19,20,23-25,27,29-32,35,38,39,41,42,50-52], [55-59], 15 case series [16,26,33,34,36,37,45,47,53,60-65], 6 phase II studies [18,40,46,48,49] and 4 pediatric studies [28,43,44,54] where Imatinib was used for the treatment of patients with DFSP. These studies include a total of 199 patients. Additional files 2, 3, 4 and 5 provide a summary of these studies including case reports (Additional file 2), case series (Additional file 3), phase II studies (Additional file 4) and pediatric reports/series (Additional file 5). A phase II study of malignancies associated with Imatinib sensitive tyrosine kinases, by Heinrich et al. was excluded. A subset of patients with DFSP in this study were further analyzed and reported separately by McArthur et al. [40,67].

All except one case report described some response to Imatinib [38]. Since the response assessment criteria varied in many case series and to minimize bias, only phase II studies were used for calculation of percentage response to Imatinib. Overall combined response rate (Complete, partial or stable disease) was 65% (48/74 patients) in phase II studies. Common indications for use of Imatinib in the selected studies included locally advanced primary, primary tumor in a cosmetically sensitive location, locally recurrent tumors, positive margins or metastatic disease. Commonly used dosages include 400 mg/day, 400 mg twice daily or 800 mg/day. Some studies utilized a dose escalation strategy where patients were started on 400 mg/day and dose was gradually increased to 600 mg/day and then 800 mg/day based on tolerance and side effects. Others utilized a higher dose of 400 mg twice daily or 800 mg/day and decreased the dose depending on the side effects. Duration of adjuvant strategy varied from 2 months to 2 years whereas neoadjuvant treatment was guided by response of the tumor to the therapy.

## Discussion

To our knowledge, this is the first case report where an unresectable pancreatic metastasis was treated in a neoadjuvant fashion with Imatinib and an R0 resection was successfully performed. Consistent with findings observed in neoadjuvant therapy for primary cutaneous disease, when a clinical and radiographic reduction in tumor size is observed, it corresponds with the histologic findings of markedly decreased cellularity as evidenced by fewer CD34+ cells, along with significant hyalinization of dermal collagen as seen in our case (Figure 5A-C). In the current case, neoadjuvant Imatinib resolved the presenting symptoms (GI bleeding, anemia, and pain) and enabled R0 resection of an unresectable metastasis. The dose of 400 mg BID was selected based on the only published phase II study, B2225 at that time in 2007 [40]. This study reported a 100% response rate in eight locally advanced t (17;22) positive DFPS tumors with four patients (50%) exhibiting complete responses. Additionally McArthur et al. reported 7/8 (88%) patients tolerated the therapy with only one patient requiring dose reduction to 600 mg. Since B2225 three additional phase II trials (Additional file 4) have been conducted evaluating 400 mg, 600 mg and 800 mg doses which have reported similar efficacy and tolerance profiles [46,49].

In this case 400 mg BID Imatinib followed by resection facilitated excellent local control evident now 5 years post metastatectomy, 10-years from primary cutaneous diagnosis without resection bed recurrence. Although, the patient did develop distant, para-aortic nodal metastasis 14 months post-resection, metastatic disease remained Imatinib responsive and demonstrated radiographic resolution with therapy. Importantly the patient did not report any significant gastrointestinal or other side effects during neoadjuvant therapy or subsequent therapy for nodal recurrence.

## Conclusions

In conclusion, given the intrinsic biological sensitivity of COL1A1/PDGFB positive DFSP to Imatinib, neoadjuvant therapy with this medication may not only be an important tool in managing locally advanced and recurrent cutaneous disease but equally valuable in unresectable or marginally resectable metastatic DFSP; facilitating margin negative resection, improving local control, and extending disease free and overall survival. The response observed following neoadjuvant Imatinib may also be useful as an in vivo test of tumor’s responsiveness and may be useful in determine the best post-operative adjuvant therapy. Neoadjuvant and adjuvant Imatinib is well established in Gastrointestinal Stromal Tumors (GIST), unlike in DFSP. The results from the closed clinical trial NCT00243191/SARC004 will provide insight into the value of short course therapy in cutaneous disease but additional trials are needed to address the value in the setting of metastatic DFSP. This report highlights the value of neoadjuvant Imatinib in facilitating surgical resection and the continued response of distant disease sites in a patient now over 5-years with metastatic DFSP.

### Consent

“Written informed consent was obtained from the patient for publication of this Case Report and any accompanying images. A copy of the written consent is available for review by the Editor-in-Chief of this journal.”

## Abbreviations

COLA1A1: Collagen 1 alpha 1; DFSP: Dermatofibrosarcoma protuberans; DFSP- FS: Dermatofibrosarcoma protuberans – fibrosarcomatous type; NED: No evidence of disease; PDGFB: Platelet derived growth factor beta.

## Competing interest

The authors declare that they have no competing interest.

## Authors’ contributions

MD – Conception and design, analysis and interpretation of the data, drafting and critical review of manuscript, final approval of the manuscript. DGC – conception, data acquisition, and final approval of manuscript. TMS & WJH - conception, data acquisition, critical review and final approval of manuscript. PTS - data acquisition, critical review and final approval of manuscript. JMF - Conception and design, analysis and interpretation of the data, drafting and critical review of manuscript, final approval of the manuscript.

## Supplementary Material

Additional file 1 Summary of studies of pancreatic metastases in patients with Dermatofibrosarcoma Protuberans. Click here for file

Additional file 2 Summary of case reports utilizing Imatinib for the treatment of patients with Dermatofibrosarcoma Protuberans. Click here for file

Additional file 3 Summary of case series utilizing Imatinib for the treatment of patients with Dermatofibrosarcoma Protuberans. Click here for file

Additional file 4 Summary of phase II studies utilizing Imatinib for the treatment of patients with Dermatofibrosarcoma Protuberans. Click here for file

Additional file 5 Summary of the case reports and series utilizing Imatinib for the treatment of pediatric patients with Dermatofibrosarcoma Protuberans. Click here for file
